# No bidirectional relationship between constipation and colorectal cancer in European and Asian populations: A Mendelian randomization study

**DOI:** 10.1097/MD.0000000000040206

**Published:** 2024-10-25

**Authors:** Ailikamu Aierken, Yierzhati Aizezi, Falide Atabieke, Mayinuer Rehaman, Munire Aierken, Shui-Xue Li

**Affiliations:** a Graduate School of Xinjiang Medical University, Urumqi, Xinjiang Uygur Autonomous Region, China; b Department of General Surgery, Children’s Hospital of Xinjiang Uygur Autonomous Region, Urumqi, Xinjiang Uygur Autonomous Region, China; c Department of Critical Care Medicine, First Affiliated Hospital of Xinjiang Medical University, Urumqi, Xinjiang Uygur Autonomous Region, China; d The second Department of Gastroenterology, The First Affiliated Hospital of Xinjiang Medical University, Urumqi, Xinjiang Uygur Autonomous Region, China; e Department of Physiology, School of Basic Medical Sciences, Xinjiang Medical University, Urumqi, Xinjiang Uygur Autonomous Region, China; f Department of Disinfection and Vector-Borne Pathogen Control, Urumqi City Center for Disease Prevention and Control, Urumqi, Xinjiang Uygur Autonomous Region, China.

**Keywords:** causality, colorectal cancer, constipation, Mendelian randomization analysis

## Abstract

Traditional observational studies have reported a positive association between constipation and the risk of colorectal cancer (CRC). However, evidence from other approaches to pursue the causal relationship between constipation and CRC is scarce. In the study, 2-sample Mendelian randomization analysis was conducted to investigate the potential causal relationship between constipation and CRC. Analysis of the results showed that there was no causal association between constipation and CRC, either in European populations (CRC: odds ratio [OR] = 1.00, 95% confidence interval [CI] = 0.99–1.00, *P* = .49; rectal cancer: OR = 0.99, 95% CI = 0.99–1.00, *P* = .79) or in Asian populations (CRC: OR = 1.00, 95% CI = 0.99–1.01, *P* = .30). Also there was no inverse causal association between CRC and constipation, either in European populations (CRC: OR = 0.10, 95% CI = 2.76E-03–3.45, *P* = .20; rectal cancer: OR = 0.05, 95% CI = 9.14E-07–2.64E + 03, *P* = .59) or in Asian population (CRC: OR = 1.18, 95% CI = 0.92–1.52, *P* = .20), there was no horizontal diversity in the instrumental variables in the Mendelian randomization analyses of the present study (all *F* statistics >10), and no heterogeneity was found in the regression analyses. The findings from bidirectional 2-sample Mendelian randomization analyses indicate that there is no evidence of a bidirectional causal association between constipation and CRC. However, further investigation is warranted through additional clinical studies and trials to thoroughly explore the association between these 2 factors.

## 1. Introduction

A few decades ago, colorectal cancer (CRC) was seldom diagnosed, but presently, it ranks as the fourth deadliest cancer worldwide, contributing to nearly 900,000 deaths annually.^[1]^ Over the past decade, successful implementation of effective CRC screening programs has led to a relatively stable or declining CRC incidence in high-income countries, whereas in low and middle-income nations, the incidence of CRC continues to escalate.^[2]^ It is now firmly established that population aging, shifts in dietary habits, and unfavorable risk factors such as obesity, insufficient physical activity, and smoking contribute to an elevated risk of CRC.^[3]^ From a clinical perspective, the early detection and diagnosis of CRC pose challenges in the absence of validated clinical features, unless proactive early screening is conducted. Research indicates that abdominal pain, changes in bowel habits, and rectal bleeding are the 3 most prevalent symptoms.^[4]^ In the event of these symptoms, seeking prompt medical attention is imperative to promptly rule out the possibility of CRC.

Constipation is a common symptom that can lead to disruptions in defecation patterns. These alterations may manifest as challenges in passing stool, excessive straining during bowel movements, a sensation of anorectal obstruction, and a feeling of incomplete evacuation. Several studies have reported a global prevalence of constipation at approximately 15%, and it is regarded as one of the most frequently diagnosed gastrointestinal conditions in medical clinics.^[5]^ As constipation can induce changes in bowel habits, it may indirectly contribute to the development of CRC. Furthermore, constipation has been linked to various diseases, including metabolic disorders, neurogenic conditions, and colorectal ailments.^[6]^ Consequently, a connection between constipation and CRC has been established. Extensive research in the past four decades has included numerous animal models and clinical studies. However, the findings have exhibited considerable variation, rendering the association between constipation and CRC risk controversial. Several case-control, cohort, and cross-sectional studies have been conducted to investigate this matter. Nevertheless, it is crucial to acknowledge that these study designs can only demonstrate associations and are unable to establish causation. Additionally, case-control studies are prone to confounding factors and the potential for reverse causality, inherent limitations that cannot be entirely mitigated.

Mendelian randomization (MR) has emerged as a valuable approach for analyzing and assessing the connection between observed modifiable exposures or risk factors and clinical risks. MR uses genetic variants as instrumental variables to infer the causal relationship between a risk factor (such as constipation) and an outcome (such as CRC). Since genetic variants are randomly inherited and fixed at conception, MR helps to reduce confounding and reverse causality, providing more robust evidence of causality compared to traditional observational studies.^[7]^ In this study, we utilized a 2-way 2-sample MR analysis to explore the potential causal relationship between constipation and the risk of CRC. This approach provides an effective alternative for evaluating causality in our research.

Biologically, several mechanisms could potentially link constipation and CRC. Chronic constipation may lead to prolonged contact between the colonic mucosa and carcinogens present in the stool, increasing the risk of colorectal inflammation and mutations. Additionally, constipation-related changes in gut microbiota may influence carcinogenesis by promoting pro-inflammatory pathways and altering bile acid metabolism. These mechanisms suggest that constipation could contribute to CRC development, further warranting exploration in our study.

## 2. Materials and methods

### 2.1. Data sources and statistical summaries

The constipation statistics were derived from 2 distinct biobanks: the Finnish Biobank, which is a database encompassing the European population, and the Japanese Biobank, which focuses on the Asian population. The Finnish Biobank dataset consisted of 342,499 cases, including 30,490 cases and 312,009 controls, and encompassed a total of 16,380,466 single nucleotide polymorphisms (SNPs). On the other hand, the Japanese Biobank dataset comprised 176,629 cases, including 397 cases and 176,232 controls, and encompassed a total of 134,358,515 SNPs. The data concerning CRC were obtained from the Gene ATLAS database (http://geneatlas.roslin.ed.ac.uk/) and KoGES PheWeb (https://koges.leelabsg.org/phenotypes). The Gene ATLAS database is an open access database with 452,264 UK Biobank samples, while the KoGES PheWeb database is an open access database with 239,989 Asian biobank samples, including 8545 cases and 231,444 controls. In the genome-wide association study of exposure and outcome, there was no population overlap between exposure and outcome. The basic information and data extraction of the included studies are shown in STable 1, Supplemental Digital Content, http://links.lww.com/MD/N785.

### 2.2. Mendelian randomized design

**Figure 1.**
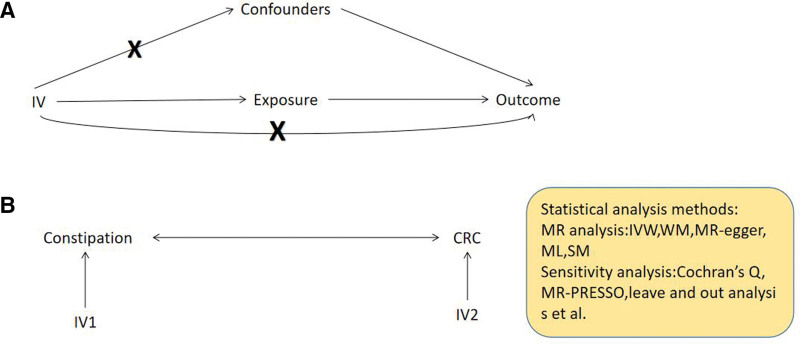
Design diagram of MR. The basic principles of 2-sample Mendelian randomization study. (A) The 3 principle assumptions. (B) The bi-directional MR design. CRC = colorectal cancer, IV = instrumental variable, IVW = inverse variance weighted, ML = maximum likelihood, MR = Mendelian randomization, SM = simple median, WM = weighted median.

The design of MR studies with constipation as an instrumental variable is based on 3 key assumptions: The instrumental variable must be strongly associated with constipation; the instrumental variable cannot be associated with confounders; the instrumental variable can lead to CRC only through its effect on constipation. The design of MR studies with CRC as an instrumental variable is based on 3 key assumptions: The instrumental variable must be strongly associated with CRC; the instrumental variable cannot be associated with confounders; the instrumental variable can lead to chronic constipation only through its effect on CRC,^[8,9]^ as detailed in Figure 1.

### 2.3. Selection of instrumental variables

First, SNPs were used as instrumental variables to identify SNPs that were strongly associated with exposure by SNPs (*P* < 5 × 10^−8^). This threshold was chosen because it represents genome-wide significance, minimizing the likelihood of false positives. However, due to the limited number of SNPs reaching this strict threshold, more relaxed *P* value thresholds (*P* < 1 × 10^−5^) were also used to include additional SNPs that may still be relevant to the exposure but fall short of genome-wide significance. These relaxed thresholds allow us to increase the pool of potential instrumental variables, which is particularly useful when the number of genome-wide significant SNPs is insufficient for robust analysis. Second, to obtain independent SNPs, the SNPs that were highly correlated with the exposure factors and without linkage disequilibrium *r*^2^ < 0.001 and kb >10,000, were selected based on the Human Genome Project’s reference data for European populations versus the reference genome data for Asian populations.^[10]^ Third, the strength of each instrumental variable was examined by calculating the *F* statistic (*F* = *R*^2^ × (*N* – 2)/(1 – *R*^2^)), where an *F* value exceeding 10 signifies the absence of weak instrumental variable bias. The *F* statistic threshold of 10 is commonly used in MR studies to ensure that the instrumental variables are sufficiently strong. An *F* statistic below 10 indicates a higher risk of weak instrument bias, which can compromise the validity of the causal inference. This threshold is supported by empirical evidence, showing that values greater than 10 provide robust instrumental variables, thus minimizing bias in the estimates. When constipation was used as an instrumental variable, N was the sample size of constipation patients, and *R*^2^ represented the proportion of constipation variation explained by each selected SNPs,^[11,12]^ and when CRC was used as an instrumental variable, N was the sample size of CRC patients, and *R*^2^ represented the proportion of CRC variation explained by each selected SNPs, SNPs with an *F* statistic of greater than 10 were selected to prevent instrumental variable bias. Fourth, the screened SNPs were evaluated in PhenoScanner V2 for the presence of confounders. If confounders existed, they were excluded. Fifth, SNPs that were directly and significantly associated with the results (*P* < 5 × 10^−6^) were finally excluded in order to fulfill the exclusivity assumption.

### 2.4. Ethical audit

Relevant data were obtained from FinnGen (https://www.finngen.fi/en), Genome-Wide Association Studies (GWAS) summary data (https://gwas.mrcieu.ac.uk/), BBJ (https://pheweb.jp/downloads), and KoGES PheWeb (https://koges.leelabsg.org/phenotypes) for publicly available data, informed consent and ethical approval was obtained from the original publications and these publicly available databases before conducting the study, which utilized the GWAS summary statistics to include SNPs associated with CRC and constipation in European and Asian pedigrees to be explored without the need for intramural ethical approval.

### 2.5. MR analysis and data visualization

Data were statistically analyzed using R4.3.0 software and R packages (TwoSampleMR, MR-PRESSO). The summary statistics of the datasets on exposure factors and clinical outcomes were harmonized such that the effect of SNPs on exposure factors and the effect of SNPs on clinical outcomes corresponded to the same alleles, respectively. Five methods, including MR inverse variance weighted (IVW), maximum likelihood, MR Egger regression, weighted median, and simple median, were applied to perform bidirectional 2-sample MR analyses, respectively, and to visualize the above odds ratios (ORs) and 95% confidence intervals (CIs) of the 5 methods.

### 2.6. Sensitivity analysis

To clarify whether there were differences between individual SNPs, heterogeneity was analyzed using Cochran’s *Q* test. If a *P* value of <.05 was obtained, heterogeneity existed, and if heterogeneity existed, a multiplicative random-effects model was used. The MR Egger intercept test was employed to evaluate the existence of horizontal pleiotropy. A deviation of the intercept from the origin suggests potential horizontal pleiotropy. The presence of horizontal pleiotropy in the instrumental variables indicates that the outcome itself may be present even in the absence of exposure factors. Leave-one-out (LOO) analysis was used to stepwise exclude each SNPs for sensitivity analysis. Finally, stability was determined by looking at the funnel plot results, as well as using the MR-PRESSO method to identify outliers and evaluate the impact of outliers on the results.

## 3. Results

### 3.1. Screening of genetic instrumental variables

Using constipation as an exposure factor and CRC as an outcome variable, 54 SNPs were obtained as instrumental variables associated with constipation, all of which met the significance threshold for genome-wide exposure (*P* < 1 × 10^−6^, *r*^2^ < 0.001, kb = 10,000). One SNP (rs1916295) was excluded in order to eliminate confounders associated with body mass index (STable 2, Supplemental Digital Content, http://links.lww.com/MD/N785). No outlier SNPs were found in any of the outlier tests in the MR-PRESSO model. In addition, the estimates of *F* statistics indicated that there were no weak instrumental variables in the MR analyses of the present study (all *F* statistics >10) (STable 2, Supplemental Digital Content, http://links.lww.com/MD/N785).

Using CRC as an exposure factor and constipation as an outcome variable, 80 SNPs were obtained as instrumental variables related to CRC, all of which met the significance threshold for genome-wide exposure (*P* < 1 × 10^−5^, *r*^2^ < 0.001, kb = 10,000) (STable 3, Supplemental Digital Content, http://links.lww.com/MD/N785). No confounders were seen based on the PhenoScanner database. No outlier SNPs were found in any of the outlier tests in the MR-PRESSO model. In addition, the estimates of *F* statistics indicated that there were no weak instrumental variables in the MR analyses of this study (all *F* statistics >10) (STable 3, Supplemental Digital Content, http://links.lww.com/MD/N785).

### 3.2. Causal effects of constipation on CRC

**Figure 2.**
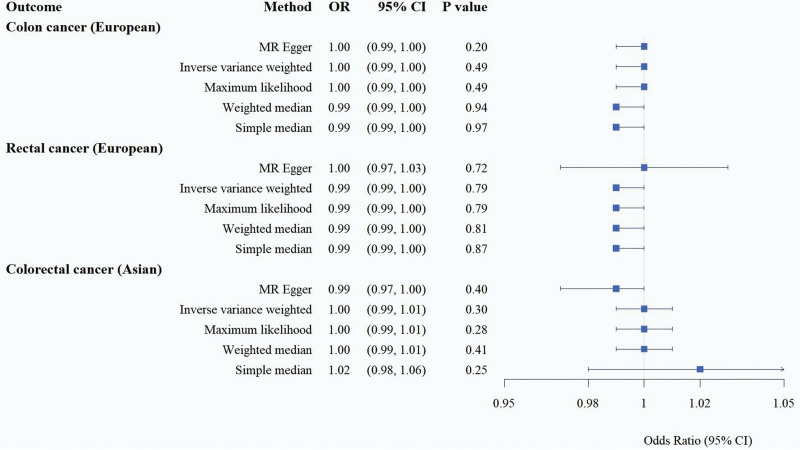
Forest plot of the causal effect of constipation on CRC. CI = confidence interval, CRC = colorectal cancer, MR = Mendelian randomization, OR = odds ratio.

**Figure 3.**
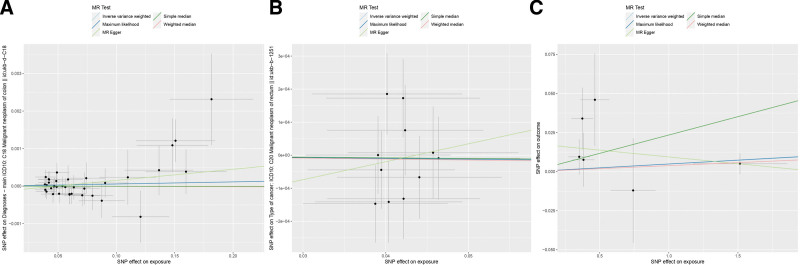
Scatter plot of 2-sample MR analysis. Mendelian randomization estimation plot showing the causal relationship between constipation and CRC. Five different methods (IVW, MR-Egger, maximum likelihood, weighted median, simple median) were used. (A) Scatterplot of constipation-associated SNPs against colon cancer risk in a European population; (B) scatterplot of constipation-associated SNPs against rectal cancer risk in a European population; (C) scatterplot of constipation-associated SNPs against CRC risk in an Asian population. CRC = colorectal cancer, IVW = inverse variance weighted, MR = Mendelian randomization, SNPs = single nucleotide polymorphisms.

There was no horizontal heterogeneity in the instrumental variables, and MR analysis was performed with constipation as an exposure factor and CRC as an outcome, and the results of the 5 methods suggested that there was no causal association between constipation and the increased risk of CRC at *P* > .05. Considering that the level of diagnosis, treatment, and prevention of constipation may be related to each geographical area, subgroup analysis of populations was conducted, and the results of IVW random-effects model were used as an example: the European population: (CRC: OR = 1.00, 95% CI = 0.99–1.00, *P* = .49; rectal cancer: OR = 0.99, 95% CI = 0.99–1.00, *P* = .79). The Asian population results were also the same: (CRC: OR = 1.00, 95% CI = 0.99–1.01, *P* = .30). It can be assumed that constipation has no positive causal effect on CRC (Table 1, Figs. 2 and 3).

**Table 1:** MR estimates of the relationship of genetically predicted constipation on CRC(*P* < 1 × 10^−5^).

Outcome	Method	OR	95% CI	*P* value	Cochran *Q* (*P* value)	MR-Egger intercept (*P* value)
Colon cancer (European population)	MR Egger	1.00	0.99-1.00	.2		.26
	Inverse variance weighted	1.00	0.99–1.00	.49	.91	
	Maximum likelihood	1.00	0.99–1.00	.49		
	Weighted median	0.99	0.99–1.00	.94		
	Simple median	0.99	0.99–1.00	.97		
Rectal cancer(European population)	MR Egger	1.00	0.97–1.03	.72		.71
	Inverse variance weighted	0.99	0.99–1.00	.79	.44	
	Maximum likelihood	0.99	0.99–1.00	.79		
	Weighted median	0.99	0.99–1.00	.81		
	Simple median	0.99	0.99–1.00	.87		
Colorectal cancer (Asian population)	MR Egger	0.99	0.97–1.00	.40		.17
	Inverse variance weighted	1.00	0.99–1.01	.30	.33	
	Maximum likelihood	1.00	0.99–1.01	.28		
	Weighted median	1.00	0.99–1.01	.41		
	Simple median	1.02	0.98–1.06	.25		

CI = confidence interval, CRC = colorectal cancer, MR = Mendelian randomization, OR = odds ratio.

Heterogeneity among instrumental variables was calculated by the IVW method, and the results of each data group did not show significant heterogeneity (Table 1). Outlier SNPs were not found by the outlier test in the MR-PRESSO model, and the test of horizontal pleiotropy by the Egger intercept method showed that the instrumental variables did not significantly affect the outcome through pathways other than exposure (Table 1). Sensitivity analyses by LOO method showed stable results (Figure S1, Supplemental Digital Content, http://links.lww.com/MD/N784). In addition, the conclusions of IVW analyses were validated by the weighted median and simple median methods, which also showed that there was no causal association between constipation and CRC.

### 3.3. Causal effect of CRC on constipation

**Figure 4.**
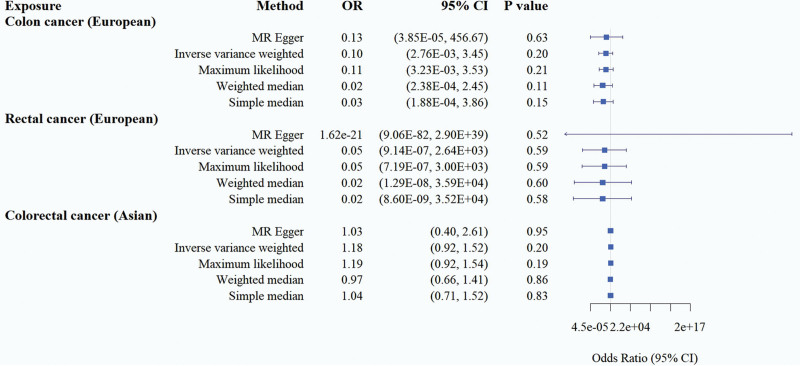
Forest plot of the causal effect of CRC on constipation. CI = confidence interval, CRC = colorectal cancer, MR = Mendelian randomization, OR = odds ratio.

**Figure 5.**
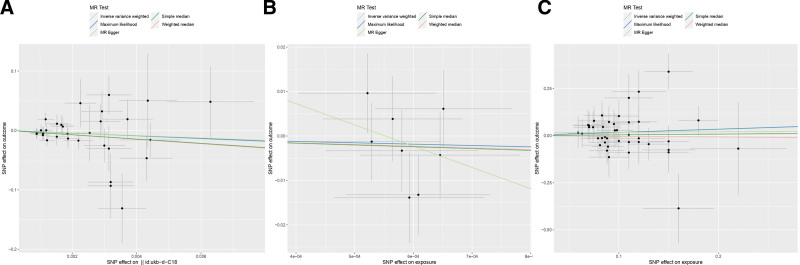
Scatter plot of 2-sample MR analysis. Mendelian randomization estimation plot showing the causal relationship between CRC and constipation. The risk log-ratio ratios are shown, using 5 different methods (IVW, MR-Egger, maximum likelihood, weighted median, simple median). (A) Scatterplot of colon cancer-associated SNPs versus constipation risk in a European population; (B) scatterplot of rectal cancer-associated SNPs versus constipation risk in a European population; (C) scatterplot of CRC-associated SNPs versus constipation risk in an Asian population. CRC = colorectal cancer, MR = Mendelian randomization, IVW = inverse variance weighted, SNPs = single nucleotide polymorphisms.

In the MR analysis with CRC as an exposure factor and constipation as an outcome, the results of the 5 methods suggested that there was no causal association between CRC and the increased risk of constipation at *P* > .05, and in the subgroup analysis with geographic targeting, no causal association was seen as well. European populations (CRC: OR = 0.10, 95% CI = 2.76E-03–3.45, *P* = .20; rectal cancer: OR = 0.05, 95% CI = 9.14E-07–2.64E + 03, *P* = .59) and Asian population: (CRC: OR = 1.18, 95% CI = 0.92–1.52, *P* = .20). It can be seen that there is also no positive causal association of CRC on constipation (Table 2, Figs. 4 and 5).

**Table 2:** MR estimates of the relationship of genetically predicted CRC on constipation (*P* < 1 × 10^−5^).

Exposure	Method	OR	95% CI	*P* value	Cochran *Q* (*P* value)	MR-Egger intercept (*P* value)
Colon cancer (European population)	MR Egger	0.13	3.85E-05–456.67	.63		.93
	Inverse variance weighted	0.10	2.76E-03–3.45	.20	.34	
	Maximum likelihood	0.11	3.23E-03–3.53	.21		
	Weighted median	0.02	2.38E-04–2.45	.11		
	Simple median	0.03	1.88E-04–3.86	.15		
Rectal cancer (European population)	MR Egger	1.62E-21	9.06E-82–2.90E + 39	.52		.54
	Inverse variance weighted	0.05	9.14E-07–2.64E + 03	.59	.55	
	Maximum likelihood	0.05	7.19E-07–3.00E + 03	.59		
	Weighted median	0.02	1.29E-08–3.59E + 04	.60		
	Simple median	0.02	8.60E-09–3.52E + 04	.58		
Colorectal cancer (Asian population)	MR Egger	1.03	0.40–2.61	.95		.76
	Inverse variance weighted	1.18	0.92–1.52	.20	.47	
	Maximum likelihood	1.19	0.92–1.54	.19		
	Weighted median	0.97	0.66–1.41	.86		
	Simple median	1.04	0.71–1.52	.83		

CI = confidence interval, CRC = colorectal cancer, MR = Mendelian randomization, OR = odds ratio.

Heterogeneity among instrumental variables was calculated by the IVW method, the results of each data group did not show significant heterogeneity (Table 1). Outlier SNPs were not found by the outlier test in the MR-PRESSO model, and the test of horizontal pleiotropy by the Egger intercept method showed that the instrumental variables did not significantly affect the outcome through pathways other than exposure (Table 1). Sensitivity analyses by LOO method showed stable results (Figure S2, Supplemental Digital Content, http://links.lww.com/MD/N784). In addition, the conclusions of IVW analyses were validated by the weighted median and simple median methods, which also showed that there was no causal association between CRC and constipation.

## 4. Discussion

Epidemiological studies have revealed that CRC accounts for 10% of all cancer diagnoses worldwide on an annual basis and contributes to 10% of cancer-related deaths.^[13]^ While the incidence of CRC among older patients remains relatively stable or shows a declining trend, there is a concerning increase in incidence rates among individuals under 50 years of age.^[14]^ Projections suggest that by 2030, approximately 11% of all CRC cases and around 23% of rectal cancers will occur in this younger age group. It is widely accepted that the majority of CRC cases are initiated by the development of polyps.^[15]^ Dysregulation of DNA repair mechanisms, prompted by various factors, leads to the formation of abnormal crypts in the colorectum. Over time, adenomas or serrated polyps progress from this initial stage, typically requiring 10 to 15 years to develop into CRC. Environmental factors, lifestyle changes^[1]^ like smoking, excessive alcohol consumption, weight gain, and increased intake of red meat, processed meat. Also, genetic factors, and intrinsic factors like obesity,^[16]^ long-term chronic inflammatory disease,^[17]^ and the onset of diabetes mellitus^[18]^ play an important role in the process of polyp and adenoma mutation. Among them, lifestyle changes are particularly close, such as antibiotic use, reduced physical activity, and obesity.^[19]^ With in-depth studies of the gut flora, it has been found that interactions between gut dysbiosis and intestinal immunity may play a key role in the pathogenesis and development of CRC,^[20]^ and that metabolites produced by the microbiota may be carcinogenic metabolites from long-term exposure, including secondary bile acids, nitrosamines, and formate. Both environmental factors and changes in lifestyle factors can affect the intestinal microflora, therefore the above factors may indirectly cause CRC by affecting the intestinal microbial population. More studies have been conducted on *Clostridium perfringens*, *Mycobacterium fragilis*, and *Escherichia coli*,^[21]^ but further research and studies are still needed to investigate how specific changes in the intestinal microbiota can affect the development of CRC and how to prevent or stop its development.

Constipation is a common gastrointestinal symptom that seriously affects the quality of human life. It can be either primary or secondary to disease or drug-induced changes. Currently, colonic sensorimotor disorders and pelvic floor dysfunction like defecation disorders are the most widely recognized causative mechanisms of constipation, and other factors, such as reduced caloric intake, microbiota disorders, altered anatomy, or pharmacological treatments, may also have an impact.^[5].^For a long time, constipation and CRC have been linked because of the similarity of their causative factors, and diet, environmental factors, and microbiota disorders play a role in the development of both. However, whether there is a direct correlation between constipation and CRC development remains uncertain. Retrospective studies have found that a greater proportion of CRC patients have a history of constipation,^[22–24]^ and thus a number of people believe that constipation increases the incidence of CRC. However, cross-sectional studies^[25,26]^ and observational studies^[27,28]^ exploring the relationship between constipation and CRC over the past decade have yielded conflicting results. Some studies suggest a positive association between constipation and CRC risk, while others do not find a significant correlation. One potential reason for this discrepancy is the inherent limitations of observational studies, including confounding factors and reverse causality. Observational studies cannot fully account for all variables that might influence both conditions, such as lifestyle factors, diet, or preexisting health conditions. In contrast, MR reduces the influence of such confounders by using genetic variants as instrumental variables, which are less likely to be affected by environmental or behavioral factors. Moreover, reverse causality, where CRC leads to constipation rather than constipation causing CRC, may explain the associations observed in some retrospective studies. Our MR analysis addresses these issues and suggests that constipation is unlikely to be a direct cause of CRC.

MR was chosen to analyze the association between constipation and CRC using MR using the publicly available GWAS database, since MR studies are less affected by confounders and exposures than observational studies and animal model studies, and because they allow the establishment of a causal association between constipation and CRC.^[29]^ Our results found no causal association between constipation and CRC in either European or Asian populations, and sensitivity analyses showed that the causal effect was not caused by outliers, level diversity. The results of the study are consistent with those of several previously conducted prospective studies.^[27,30]^ To the best of our knowledge, this is the first study to examine the causal relationship between constipation and CRC.

The relationship between constipation and CRC is established through a specific mechanism. Intestinal flora converts intestinal contents into carcinogens or co-carcinogens through fermentation. Constipation in patients poses obstacles to defecation, leading to a reduction in the number and frequency of bowel movements compared to normal individuals. This, in turn, causes the concentration of carcinogens in feces to accumulate, increasing the duration of contact between carcinogens and the colon’s mucous membranes. Consequently, there is an elevated risk of CRC.^[31]^ This theory has been corroborated by previous case–control studies. However, case–control studies have many confounding factors and cannot clarify the causal relationship between the 2, whether constipation increases the risk of CRC or CRC increases the incidence of constipation. Our findings suggest no causal association, similar to most cohort and cross-sectional studies, but previous studies have shown that dietary diets^[32]^ and the use of fiber-containing laxatives^[33–35]^ prevent CRC. High dietary fiber intake provides important beneficial metabolic substrates to colonic cells through dilution of carcinogens, binding of cancer-causing secondary bile acids, and through bacterial fermentation metabolic substrates to colonic cells and thus is associated with a reduced risk of CRC.^[32]^ In addition, dietary fiber may reduce CRC through the production of short-chain fatty acids and the normalization of cell proliferation and differentiation, which again correlates with previous theories, and more experiments are needed to elucidate the association between constipation and CRC.

The findings of our MR study, which indicate no causal association between constipation and CRC, have significant clinical implications. First, this suggests that constipation, although a bothersome and quality-of-life reducing symptom, may not need to be viewed as a contributing factor to CRC risk. This could help clinicians reassure patients that treating constipation, while important for comfort and overall health, is not expected to lower CRC risk. However, clinicians should still encourage patients to engage in lifestyle modifications, such as increasing fiber intake, physical activity, and adopting a balanced diet, as these factors independently reduce the risk of CRC. Furthermore, while constipation itself may not directly increase CRC risk, some of its underlying causes, such as low fiber intake and sedentary behavior, are well-known risk factors for CRC. Therefore, efforts to treat constipation through diet and lifestyle changes may still provide an indirect benefit in reducing CRC risk by addressing these shared risk factors. Clinicians should continue to promote a healthy lifestyle, focusing on comprehensive cancer prevention strategies.

Our findings suggest that there is no causal association between constipation and CRC. This study also has some limitations. Firstly, the genetic data used in this study came only from European and Asian populations, so its applicability may be limited by other populations. Secondly, MR studies typically lack the ability to explore biological mechanisms. While we found no genetic evidence for a causal relationship between constipation and CRC, this does not exclude the possibility that other biological pathways, such as chronic inflammation or changes in the gut microbiota, could link constipation to CRC. More research is needed to explore these mechanisms in greater detail. Finally, the data used in this study did not include information on age, sex, or other demographic factors, which limited our ability to perform subgroup analyses. Future research should aim to stratify results by demographic variables to better understand potential differences in risk among various subgroups.

## 5. Conclusion

The present study explored the association between constipation and CRC by means of a 2-sample MR study, and the results indicate that there is no causal relationship between constipation and CRC at the genetic level. This finding provides important evidence that constipation may not increase the risk of developing CRC, and similarly, CRC does not necessarily increase the incidence of constipation. However, more clinical studies and experiments are still needed to gain a more comprehensive understanding of the relationship between constipation and CRC. The results of these studies are of great significance in exploring the relationship between constipation and CRC in depth and in guiding future related research and clinical practice.

## Acknowledgments

The authors acknowledge all of the participants and investigators for contributing and sharing summary-level data on GWAS. The authors acknowledge the participants and investigators of the FinnGen study.

## Author contributions

**Data curation:** Falide Atabieke, Ailikamu Aierken, Munire Aierken.

**Formal analysis:** Falide Atabieke, Shui-Xue Li.

**Methodology:** Yierzhati Aizezi.

**Software:** Mayinuer Rehaman.

**Funding acquisition:** Shui-Xue Li.

## Supplementary Material


