# Supplementary material for: No bidirectional relationship between constipation and colorectal cancer in European and Asian populations: A Mendelian randomization study
Source: Medicine (Baltimore). 2024 Oct 25;103(43):e40206. doi: 10.1097/MD.0000000000040206 (PMC11520989; doi:10.1097/MD.0000000000040206)

Figure S1. Mendelian randomization estimation plots showing the causality of genetic prediction of constipation on colorectal cancer, with risk log-ratio ratios as shown in (A) Forest plot of constipation-associated SNPs versus colon cancer risk in European populations; (B) Forest plot of constipation-associated SNPs versus rectal cancer risk in European populations; (C) Forest plot of constipation-associated SNPs versus colorectal cancer risk in Asian populations; (D) Forest plot of constipation related SNPs on colon cancer risk; (E) forest plot of constipation-related SNPs on rectal cancer risk in European populations; (F) forest plot of constipation-related SNPs on colorectal cancer risk in Asian populations; SNP: single nucleotide polymorphism.

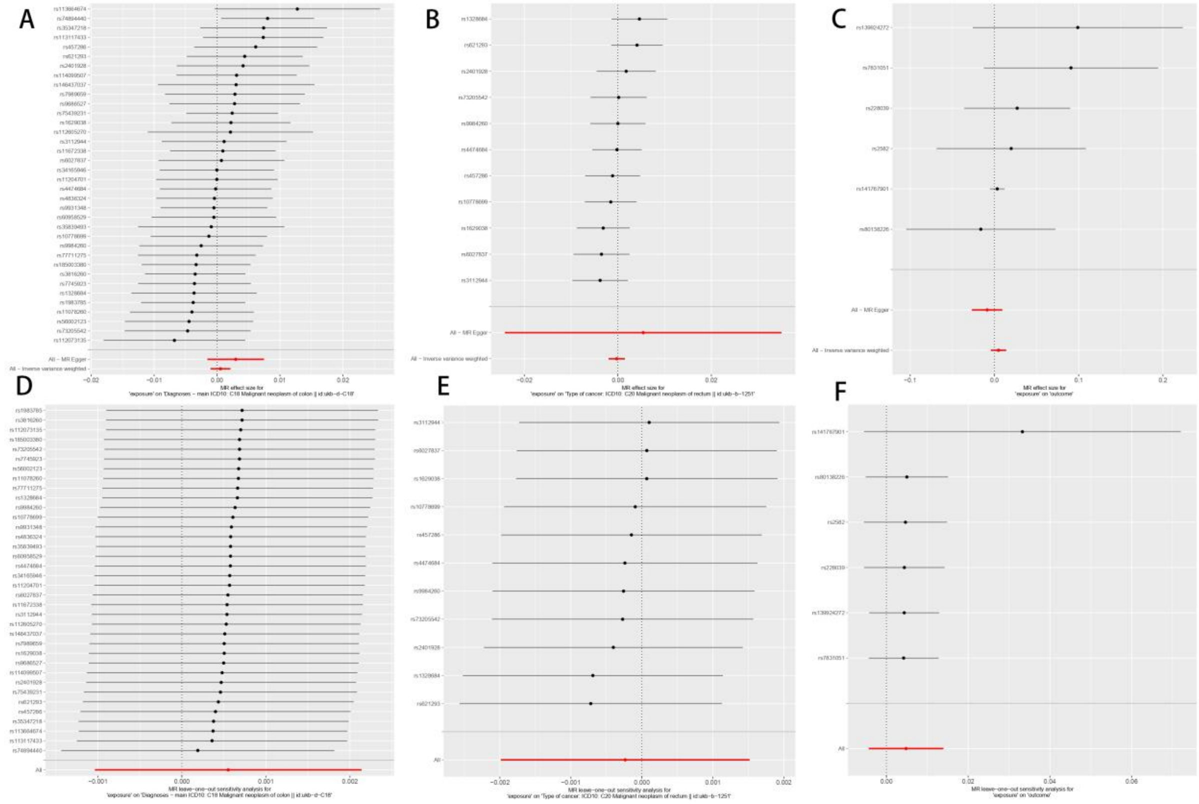

Figure S2 . Mendelian randomization estimation plots showing the causality of genetic prediction of colorectal cancer on constipation, with risk log-ratio ratios as shown in (A) Forest plot of colon cancer-associated SNPs on constipation risk in European populations; (B) Forest plot of rectal cancer-associated SNPs on constipation risk in European populations; (C) Forest plot of colorectal cancer-associated SNPs on risk in Asian populations; (D) Forest plot of colon cancer-associated SNPs on the risk of constipation; (E) Forest plot of SNPs on the risk of constipation associated with rectal cancer in European populations; (F) Forest plot of SNPs on the risk of constipation associated with colorectal cancer in Asian populations; SNP: single nucleotide polymorphism.

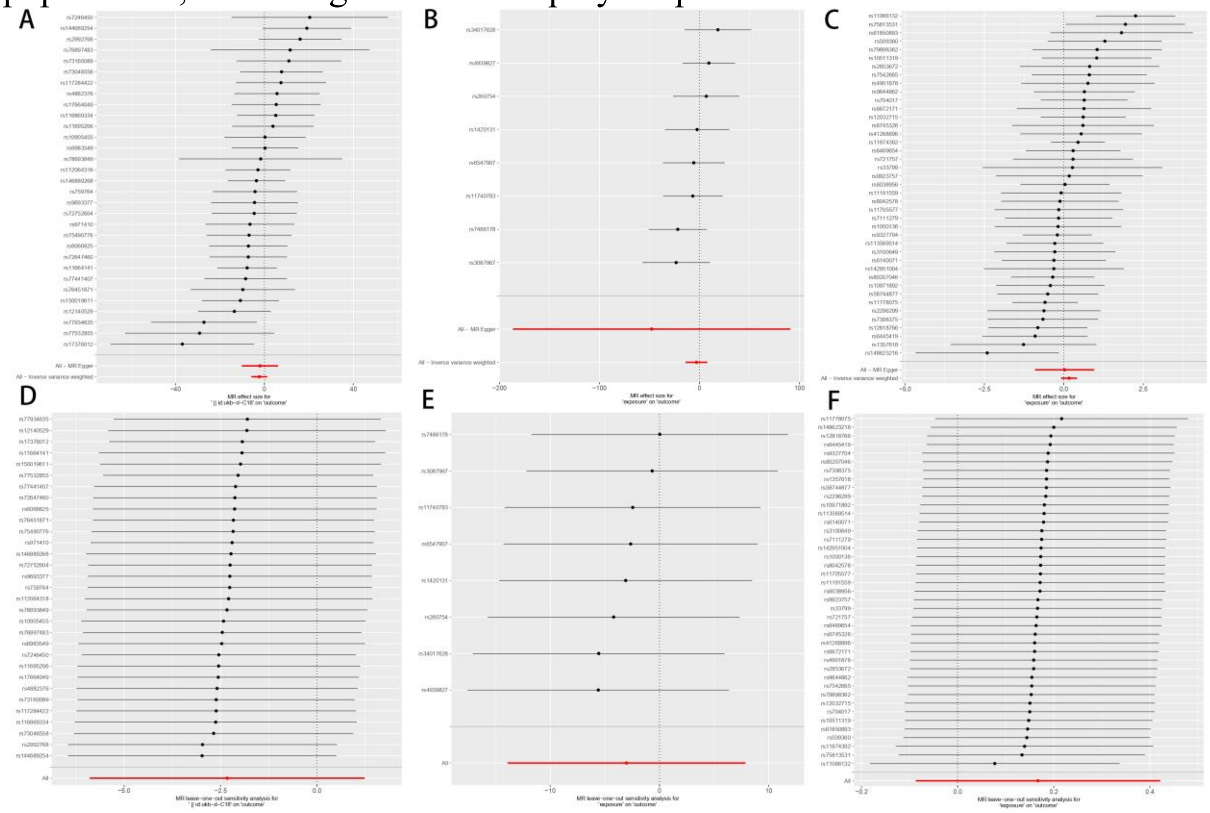

Supplement: Supplementary file 2 [file medi-103-e40206-s002.pdf]
